# Using Bayesian Dynamic Borrowing to Maximize the Use of Existing Data: A Case-Study

**DOI:** 10.1007/s43441-023-00585-3

**Published:** 2023-11-01

**Authors:** Dawn Edwards, N. Best, J. Crawford, L. Zi, C. Shelton, A. Fowler

**Affiliations:** 1 GSK, 980 Great West Road, Brentford, TW8 9GS Middlesex UK; 2 GSK, Shanghai, China; 3 GSK, Upper Providence, PA USA

**Keywords:** Bayesian dynamic borrowing, Case-study, Clinical study data re-use, China

## Abstract

Bayesian Dynamic Borrowing (BDB) designs are being increasingly used in clinical drug development. These methods offer a mathematically rigorous and robust approach to increase efficiency and strengthen evidence by integrating existing trial data into a new clinical trial. The regulatory acceptability of BDB is evolving and varies between and within regulatory agencies. This paper describes how BDB can be used to design a new randomised clinical trial including external data to supplement the planned sample size and discusses key considerations related to data re-use and BDB in drug development programs. A case-study illustrating the planning and evaluation of a BDB approach to support registration of a new medicine with the Center for Drug Evaluation in China will be presented. Key steps and considerations for the use of BDB will be discussed and evaluated, including how to decide whether it is appropriate to borrow external data, which external data can be re-used, the weight to put on the external data and how to decide if the new study has successfully demonstrated treatment benefit.

**Supplementary Information:**

The online version contains supplementary material available at 10.1007/s43441-023-00585-3.

## Introduction

Leveraging external data when designing a new clinical study can enhance the information content of that study or the overall development program. The objective of using external data could be extrapolation of efficacy from one population to another, or to use data on placebo or standard of care from other studies in the analysis of future studies. Hence, the aim is either to:

- Reduce the sample size required to achieve a desired level of confidence for the new study.
- Boost its decision-making power without large increases in sample size.

Traditionally, existing data are used to inform the *design* of subsequent studies (e.g., sample size estimation). However, incorporating existing data into the *analysis* of a new study is less common.

One way to incorporate existing data are through a method known as Bayesian Dynamic Borrowing (BDB), an approach with increasing use in clinical development. The regulatory acceptability of BDB continues to evolve and experience with BDB varies between, and within, regulatory agencies [1–8].

**Figure 1:**
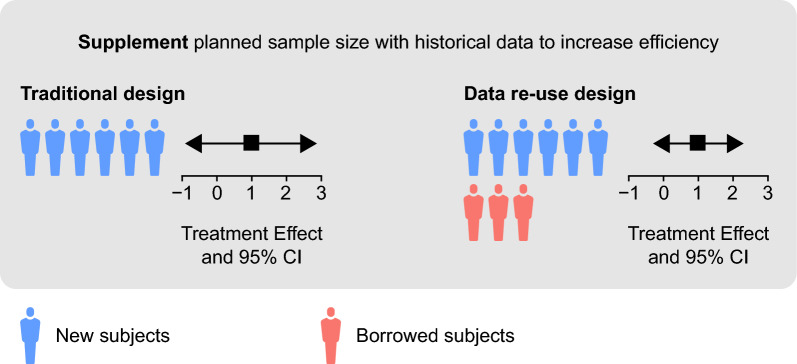
Data re-use to supplement the planned sample size. *CI* confidence interval.

This article will describe how external data can be “re-used” in a new clinical study to supplement the planned sample size (Fig. 1) and discuss key considerations related to data re-use and BDB in drug development programs. A case-study illustrating the planning and evaluation of a BDB approach to support registration of a new medicine with the Center for Drug Evaluation in China (CDE) will be presented.

## How Does BDB Work?

In clinical studies, traditional (frequentist) statistical methods may use information from previous studies only at the design stage (e.g., sample size estimation). Then, at the data analysis stage, the information from these studies is considered as a complement to, but not part of, the formal analysis. In contrast, Bayesian statistics is an approach in which enables results from previous studies to directly contribute to the analysis of the data from the new study.

The Bayesian approach uses Bayes’ theorem to formally combine prior information with current information on a measure of interest such as a treatment effect [9]. “Prior information” refers to any relevant information external to the new study and does not necessarily have to be available before the study starts. The Bayesian idea is to represent this information as a probability distribution and update this “prior distribution” with the data from a new study to obtain a “posterior distribution” for the measure of interest which combines the two sources of evidence. Put another way, the Bayesian posterior borrows prior information to augment the evidence from a new study.

**Figure 2:**
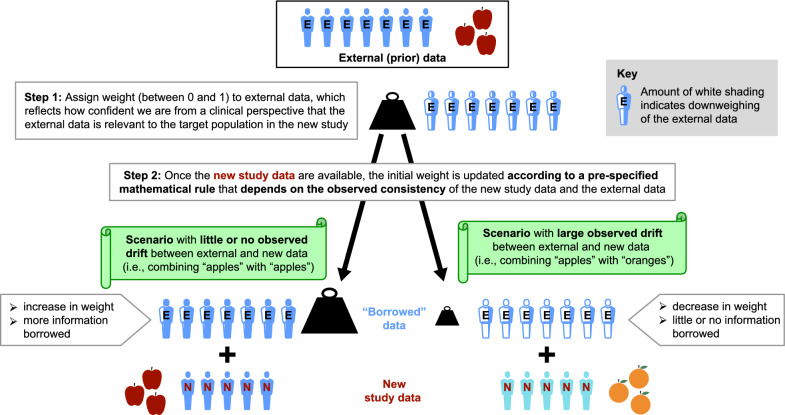
How Bayesian dynamic borrowing works.

The standard version of Bayesian borrowing assumes the measure of interest (e.g., treatment effect) in the external and new studies are “exchangeable”, i.e., similar enough to combine together. However, a key concern is that effects observed in the external data may be substantially different from the true effects in the new study. This could result in non-negligible bias in the assessment of treatment effect, leading to misguided decisions (e.g., approval of ineffective therapies or false non-approval) [10]. BDB is a particular version of a Bayesian approach designed to help mitigate the risk of borrowing inappropriate data by providing a mathematically rigorous automatic adjustment to downweight prior information that is not consistent with the new study data (Fig. 2).

## How to Plan and Evaluate a BDB Study—A Case-Study

This case-study reviews the design of a BDB study to support the approval of a drug for use in patients in China. Our approach is in line with the limited regulatory guidance currently available on design of Bayesian studies to support drug approval (e.g., FDA guidance on Complex Innovative Designs [11], draft ICH E11A guideline on Pediatric Extrapolation [12]), and is also aligned with the ICH E5 and E17 principles for bridging and multiregional trials.

A global study had been conducted to support the approval of the drug globally; however, Chinese patients were not included and therefore a separate study was required to support registration with the CDE.

The BDB approach was chosen as it utilizes the evidence already available from the global study [treatment difference 86 units (95% CI: 46, 125)] and was more likely to provide robust evidence than an unpowered standalone study.

The China BDB study was designed to be as similar as possible to the global study (for example patient inclusion/exclusion criteria, primary endpoint and estimand of interest), with the objective of assessing the effect of a new treatment compared with a reference treatment for a continuous endpoint in Chinese patients.

**Figure 3:**
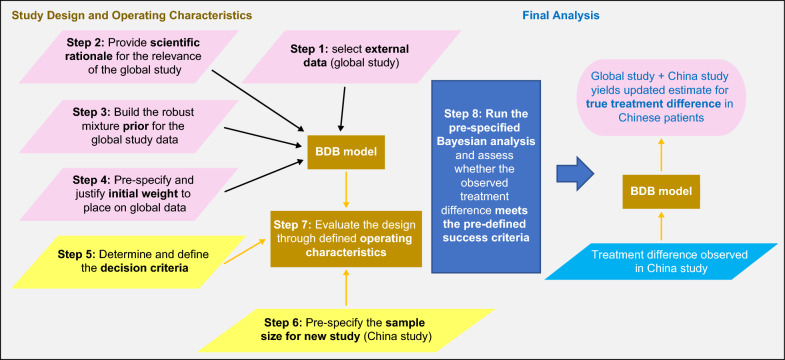
BDB bridging study approach used in this case-study. *BDB* Bayesian dynamic borrowing.

There were several steps that were followed when designing this BDB study (Fig. 3):

1. At the design stage, the **external data to borrow from** (the “prior” information) needed to be selected. In this case it was the global study. In other settings, it could be an external study or meta-analysis of several studies.
2. **Scientific rationale** for relevance of the global data were provided to support the assumption of exchangeability of the treatment effect between the global and new China studies:
  - The disease and its management are similar in Chinese and non-Chinese patients, and the countries included in the global study were considered to have similar intrinsic and extrinsic factors to China, following discussions with external experts and review of relevant data. For example, intrinsic factors such as disease pathophysiology, pharmacology including receptor expression and downstream signalling, pharmacokinetics including local drug exposure, metabolism and clearance in addition to extrinsic factors such as medical practice, drug compliance and disease definition are similar, and therefore the treatment effect should also be similar.
  - Existing data with the same drug in Chinese and non-Chinese patients for another indication with similar characteristics demonstrated a similar risk–benefit profile for the two populations.
  - Aside from ethnicity, the study designs of the global and China studies were identical in terms of inclusion/exclusion criteria, primary endpoint and estimand.
3. In order to implement BDB, a suitable method to construct a **prior** distribution for the **treatment difference** in the Chinese population using the global data needed to be chosen. In this case we used a robust mixture prior (RMP) [13] where the global data were used as an “informative” prior distribution for the treatment difference in Chinese patients. A second “vague” prior distribution was specified to allow for the possibility that the global data do not provide relevant information about the treatment difference in Chinese patients. A weighted combination of the “informative” and “vague” components was used in the RMP (Supplementary Information Section A and Supplement Fig. 1). The vague component enables the information from the global data to be downweighed in case of disparity between the observed results in the China and global populations. The weights on the “informative” and “vague” components must sum to one.
4. Pre-specify and justify the **initial weight** to place on the “informative” component of the mixture prior.
  The choice of this weight is subjective^1^ and should reflect how much scientific evidence there is to support exchangeability of the prior information (in this case, the global data) and the new study.
  The operating characteristics (OCs) of the study (Step 7) also needed to be assessed when selecting the weight to ensure that the global data does not overwhelm the data from the new study or result in unacceptable design characteristics such as large inflation of type I error (false positive rate). So, while the scientific evidence may support choosing a larger initial weight, this assessment may lead to a smaller initial weight being selected.
  In this case-study, the initial weight given to the informative component of the mixture prior was 0.3, as detailed below (and consequently, the initial weight given to the vague component was 0.7).
5. **Define the decision criteria.** Bayesian designs do not use *p*-values like a conventional frequentist study, so alternative decision criteria must be specified [11]. In this case-study, the success criterion was that the posterior probability of a true treatment benefit (i.e., true treatment difference > 0) in Chinese patients is at least 95%.
6. Pre-specify the sample size for the new China study. A range of sample sizes were explored, and a sample size of 75 patients per arm was selected based on an acceptable balance of OCs (see Step 7).
7. Evaluate the design through **defined OCs** such as power (probability of success for a given fixed value of the true treatment difference) and type I error rate, as well as assessing the impact of different choices of initial weight on the global data as well as the impact of different sample sizes.
8. Once the China study has been completed, **run the pre-specified Bayesian analysis** and assess whether the observed treatment difference **meets the pre-defined success criteria** (Step 5). This can be implemented as follows:
  Analyze the China study to obtain the mean difference and standard error for the comparison of the new and reference treatments.
  Combine this treatment difference with the pre-specified RMP based on the global study to obtain an updated (posterior) estimate for true treatment difference in Chinese patients.
9. Conduct pre-specified sensitivity analyses, including a tipping point analysis [14] to assess the robustness of conclusions to the strength of prior assumptions about similarity of the global and China study treatment effects.

**Figure 4:**
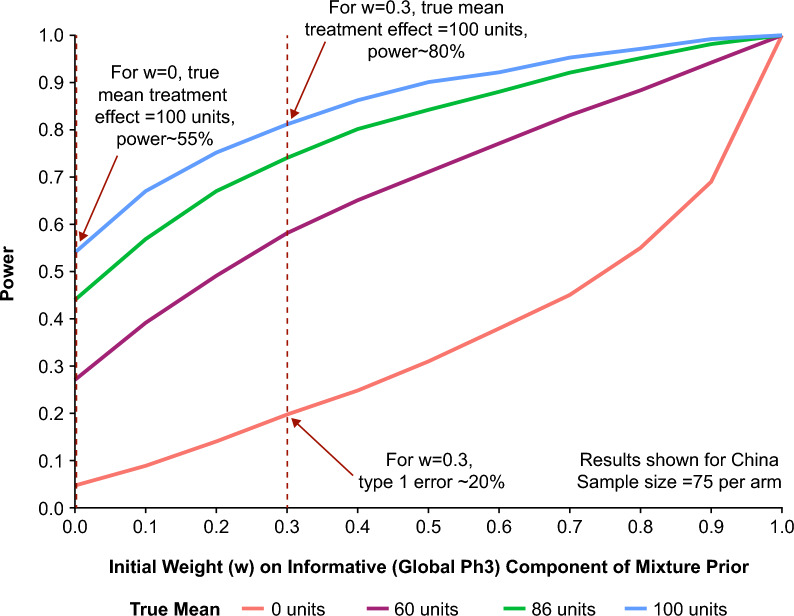
Power for new treatment versus reference treatment. *Ph3* phase 3, *W* weight.

Evaluation of OCs for BDB designs relies on complex simulations. For this case-study various plots were produced for OCs such as power and type I error rate against the prior weight on the global data. Figure 4 displays the power (probability of success conditional on different assumed true treatment difference values) based on 75 subjects per arm for the China study.

**Figure 5:**
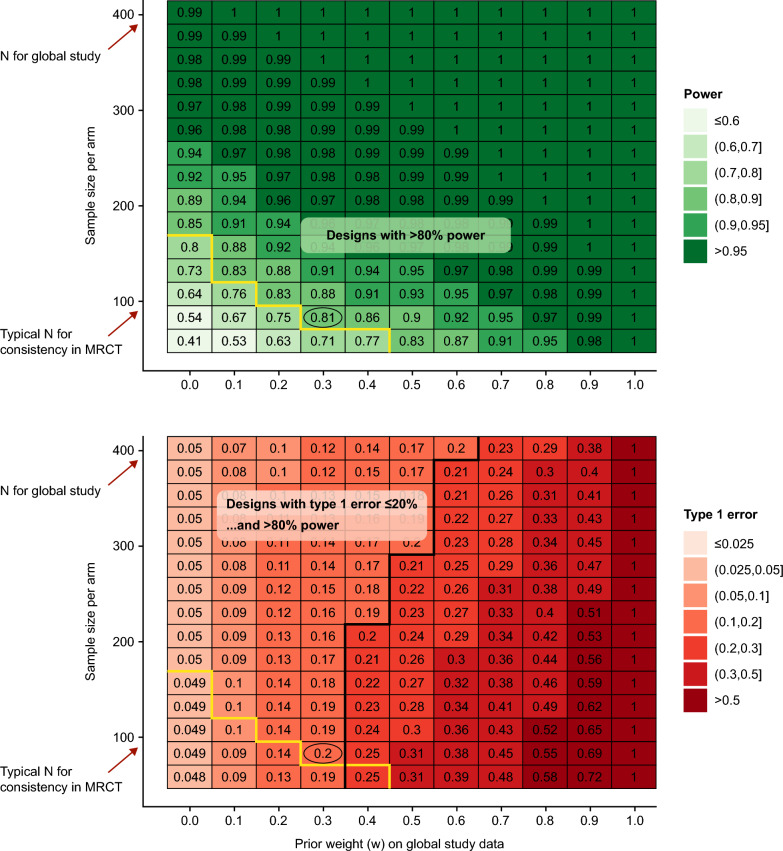
Illustration of operating characteristics to help select design parameters. Note 1: Operating Characteristics calculated using decision rule: Pr ( treatment effect > 0) ≥ 95%, Power calculated assuming true treatment effect = 100 "units”, Type I error calculated assuming true treatment effect = 0 "units”**.** Note 2: any combination of prior weight and sample size above the yellow line has power > 80% and anything to the left of the black line has type I error less than 20%. MRCT = multi-regional clinical trial.

Other graphical approaches can also be used to help select design parameters. Figure 5 presents heatmaps for both power (top heatmap) and type I error rate (bottom heatmap) to explore how changing the prior weight on the global data and sample size in the China study impacts power and type I error.

For this case-study, the sample size for the global study was approximately 400 per arm, whilst the sample size for demonstrating consistency of effect within a multi-regional clinical trial is usually around 15–20% of the overall sample size [15]. Hence it was anticipated that the target sample size for the new China study would be less than 100 patients per arm.

It can be seen from the bottom heatmap that with no borrowing of global data (i.e., prior weight = 0, equivalent to a traditional standalone study), type I error is fixed at 5%; however, the power is only 54% (top heatmap). With BDB, both type I error and power vary as a function of weight and sample size. For power (top heatmap), as prior weight on the global data increases so does the power; however, increasing the prior weight on the global data also increases type I error. Therefore, an acceptable balance of these OCs needs to be achieved. For this case-study, power of at least 80% and type I error no more than 20% were targeted to determine a range of potential sample sizes and prior weights. A prior weight of 0.3 on the global study and a sample size of approximately 75 per arm resulted in a power of approximately 80% and type I error approximately 20% (black circles in the heatmaps).

Table 1 provides more detail to support the choice of 0.3 as the initial weight to place on the global data. The ICH E11A draft guidance [12] interprets the weight on the source data in a BDB mixture prior for a pediatric extrapolation design as the prior belief about the plausibility and acceptability of the extrapolation concept. Adopting a similar interpretation here, the scientific principles to support the bridging concept between the global data and the new China study justified a high initial weight (~ 0.7–0.8) on the global data. However, when leveraging external data in a Bayesian design, ICH E11A draft guidance also recommends use of OC simulations to inform analysis choices with a view to optimizing the trade-off between bias, power and type I error rate. Therefore, based on the OCs of the BDB design summarised above, a more conservative prior weight of 0.3 was felt to provide an acceptable trade-off between the risk of a false positive result and a high power, while ensuring that the global data does not overly influence the data from the new study but allows the observed data in Chinese patients to contribute to the inference from the study.

**Table 1:** Summary of power and type I error rate when initial weight on the global data are 0.3

Operating Characteristic	Value	Commentary
Probability of a false positive result (assuming true treatment difference = 0 units)	20% (Type I error rate)	Increasing weight further would increase POS (type I error) above 20% As the prior weight increases so does the type I error—however, in bridging settings, there already is good prior evidence of treatment benefit, so the risk of committing a type I error is low because likelihood that treatment is truly ineffective is low. A type I error of 20% for this case-study seemed reasonable
Probability of true positive result (assuming true treatment difference = 100 units)	81% (Power)	If the true mean difference is 100 units (which is around what was believed to be the true treatment difference based on the global study), then the POS is 81% and increasing weight has less significant impacts in improvements in POS further (i.e. the increase in POS plateaus)

Note 1: Results based on using robust mixture prior with initial weight 0.3 on the global data which had an estimated treatment effect of 86 units

Other OCs of interest were also examined and similar plots to Fig. 4 were produced and detailed in the supplement (Online Resource).

Table 2 provides a summary of the posterior distribution of the true treatment difference (based on a prior weight of 0.3 on the global data) for a range of scenarios each representing a hypothetical outcome (i.e., value of the observed treatment difference) in the China study. If the observed treatment difference in China is 49 units (which is the smallest observed value needed to meet the success rule of providing at least 95% probability that the true China treatment difference is > 0), the point estimate (posterior mean) of the true treatment difference in China based on the posterior weighted combination of the global and China data are 73 units, with 90%^2^ credible interval (> 0, 118). In contrast, for the scenario of no borrowing of the global data (prior weight = 0), the smallest observed value needed in the China study to meet the success rule would be almost 100 units (Online Resource: Supplement Fig. 6). The probability of meeting the study success criteria in this second scenario is low.

**Table 2:** Summary of the posterior distribution of true treatment effect given a range of hypothetical outcomes (Observed Treatment Differences) in the China Study

Hypothetical outcome (Observed treatment difference) in China Bridging study	Updated (posterior mean) estimate of true treatment difference in China	90% Credible Interval for true treatment difference in China
0 units	43 units	(– 68 units, 105 units)
49 units	73 units	(> 0 units, 118 units)
60 units	77 units	(14 units, 121 units)
86 units	86 units	(38 units, 132 units)
100 units	90 units	(46 units, 143 units)

Note 1: Results based on using robust mixture prior with initial weight 0.3 on the global data which had an estimated treatment effect of 86 units

If no difference (0 units) was observed between the treatment groups in the China study, the updated estimate and 90% credible interval for the true treatment difference in China would be 43 units (– 68, 105) and the success rule would not be met. For scenarios with higher observed treatment differences in China (60 units, 86 units or 100 units), the updated estimates of the true treatment difference in China are able to borrow more information from the global data (the updated weight on the global data increases: Online Resource: Supplement Tables 2 and 3) as there is stronger evidence of consistency between the China and global treatment effects. The greatest borrowing occurs for the scenario where the observed treatment difference in China is the same as the global study result (86 units).

Once the China study primary analysis has been run, sensitivity analyses can also be run to assess the robustness of assumptions made for the study design, in particular the choice of initial weight on the global data.

## Discussion

For this case-study, progressing with the BDB approach resulted in a greater efficiency of data use and considerable time saving, hence enabling accelerated availability of medicines to patients. An alternative, more traditional, approach conducting a standalone study in China with a trend analysis was also considered. For this approach a minimal treatment difference to observe in the bridging study is defined and success claimed if this difference is observed [15–18]. However, this difference can be rather arbitrary, such as > 0 or 50% of the global study result. The estimated treatment difference from this standalone study is used to estimate the true treatment effect; however, given the small sample size reduced precision in estimating the treatment effect is probable with a greater likelihood of erroneous conclusions. The BDB approach is more robust and generally results in greater precision of the estimate of the true treatment effect provided there is not strong evidence of inconsistency between the current study and borrowed data. In settings where BDB is used to borrow external control arm information, large inconsistencies can result in an underpowered study [16].

It may not always be appropriate to consider a BDB design (e.g., if the external data are not relevant to the new data being collected) and so careful consideration is needed, by following the steps outlined above. Also, it’s important to consider whether all of the data being used for borrowing is appropriate or whether a particular subset is more likely to be exchangeable.

There may be more of a tendency to use BDB in early phase studies where decisions are the company’s risk. However, regulators are becoming increasingly more open to the possibility of methods such as BDB in late phase studies. It is a topic of interest for several cross-industry consortia [19, 20]. The FDA recently published summaries of complex innovative design pilot studies, two of which use BDB [1, 7] and discusses some regulatory perspectives on use of BDB in pediatric clinical development [8]. Formal regulatory guidance on BDB is limited but there are increasing opportunities for Industry to discuss BDB designs with regulators as part of efforts to accelerate innovation in clinical study design (Chinadrugtrials.org.cn: identifier CTR20211487, Clinicaltrials.gov: identifier NCT05065190).

Early interaction with regulatory agencies is encouraged to ensure alignment with OCs. A larger type I error may be acceptable in situations where there is a high degree of confidence in exchangeability. For example, in the setting of pediatric extrapolation where relevant evidence of efficacy already exists in a reference population, ICH E11A draft guidance indicates that this will lead to a different relationship between the false positive rate and false negative rate than in the original reference population. For this case-study, the project team worked closely with the CDE statisticians to develop the BDB approach, ultimately resulting in CDE endorsement of the study design.

**Figure 6:**
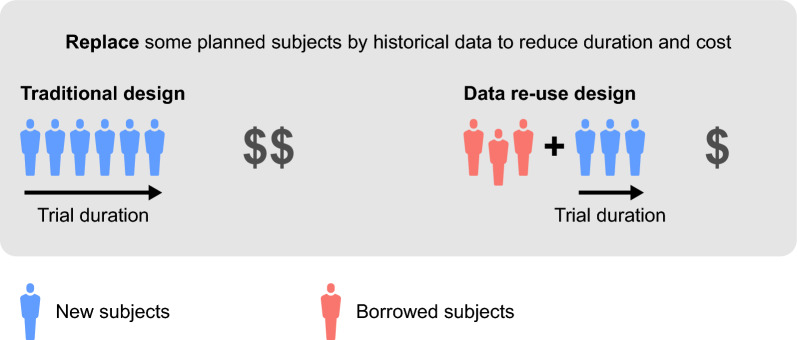
Replacement of planned subjects with historical-data.

In this case-study we borrowed information about a comparative effect for a continuous endpoint. However, it is also possible to borrow information for a non-continuous endpoint or about an endpoint directly (e.g., borrow an active or control arm) or borrow information on more than one measure in the same study. This article has focused on *supplementing* the planned sample size with external data; however, there are also methods available to enable the *replacement* of some planned subjects, hence reducing the overall sample size for a new study (Fig. 6). There are also other statistical methods available for borrowing data such as the normalized power prior and/or the commensurate prior [16]. This case-study has focused on the bridging of global datasets to a new region; alternative Bayesian approaches with informative priors could also be considered for this purpose [21–23]. Other applications of BDB could include extrapolation to pediatric populations from adult data [2–4, 8] or supplementing a study with external control data [5, 6, 24].

## Conclusion

The BDB approach covered in this paper enabled bridging of existing global data to a new region. It uses a mathematically rigorous and robust approach to integrating external data while providing a safety valve to minimize the impact of inconsistent results between the external data and new study data. This boosted the decision-making power of the China study without requiring a fully powered standalone trial, which should lead to quicker access to medicines for patients.

### Supplementary Information

Below is the link to the electronic supplementary material.

Supplementary file1 (DOCX 527 KB)

## Data Availability

Anonymized individual participant data and study documents can be requested for further research from https://www.gsk-studyregister.com/en/

## Footnotes

1 Some recent proposals attempt to *objectively* select the weight on the informative component (Egidi et al. 2021; Zhao and Ma 2023). In these methods, the prior depends on the observed data in the new study. This deviates from the traditional Bayesian workflow whereby the prior is chosen without reference to new data, and means that the prior is not known in advance (although the method for computing the prior can be fully pre-specified). As yet, there is limited experience of applying objective methods for selecting the mixture weight in practice. Egidi et al. (2021). Avoiding prior–data conflict in regression models via mixture priors. Canadian Journal of Statistics. 50. 10.1002/cjs.11637. Zhao and Ma (2023). Modified Robust Meta-Analytic-Predictive Priors for Incorporating Historical Controls in Clinical Trials. 10.1101/2023.01.28.23285146.

2 90% (equal-tailed) credible intervals are reported for consistency with the primary success criteria since at least 95% probability that the true China treatment difference > 0 is equivalent to the lower limit of the 90% credible interval > 0.
