# Supplementary material for: Using Bayesian Dynamic Borrowing to Maximize the Use of Existing Data: A Case-Study
Source: Ther Innov Regul Sci. 2023 Nov 1;58(1):1–10. doi: 10.1007/s43441-023-00585-3 (PMC10764450; doi:10.1007/s43441-023-00585-3)
Supplement: Supplementary file 1 — Supplementary file1 (DOCX 527 KB) [file 43441_2023_585_MOESM1_ESM.docx]

# Using Bayesian Dynamic Borrowing to maximise the use of existing data – a case study: Supplementary Information

Edwards D^1^, Best N^1^, Crawford J^1^, Zi L^2^, Shelton C^3^ Fowler A^1*^

^1^GSK, Brentford, Middlesex, UK; ^2^GSK, Shanghai, China; ^3^GSK, Upper Providence, PA, USA

*At the time of the study

# A: Specification of the prior distribution

A robust mixture prior [1] was used for the treatment difference (denoted $\delta$) of interest in the Chinese population, defined as a mixture of two components:

$$\begin{aligned} p\left( \delta\right)=w\cdot p_{1}\left( \delta\right)+(1-w)\cdot p_{2}\left( \delta\right) \end{aligned}$$

where

- $p_{1}(\delta)$ is the informative component based on the global study data, and is defined as $p_{1}\left( \delta\right)=Normal(m_{g}, s_{g}^{2}$) where $m_{g}=86$ and $s_{g}=20.1$ are the point estimate and associated standard error of the treatment difference in the global study;
- $p_{2}(\delta)$ is the vague component, defined as $p_{2}\left( \delta\right)=Normal(0, s_{v}^{2}$) where $s_{v}=\sqrt{2}\times SSD$ and $SSD=350$ is the sample standard deviation of the primary endpoint of interest, i.e. the vague prior has mean equal to the null hypothesis of no effect, and variance chosen so that it contains a minimal amount of information equivalent to the sampling distribution of the mean treatment difference derived from a single observation per arm;
- $w$ is the initial weight assigned to the global study information, chosen here to be 0.3

This prior is illustrated in Supplement Figure 1 below.

**Supplement Figure 1: Robust mixture prior distribution for the treatment difference in the primary endpoint**

The robust mixture prior for the China treatment effect in the bridging study combines two components: the “informative” component (derived from global study data) and the “vague” component, with pre-specified weights of 0.3 and 0.7 respectively.


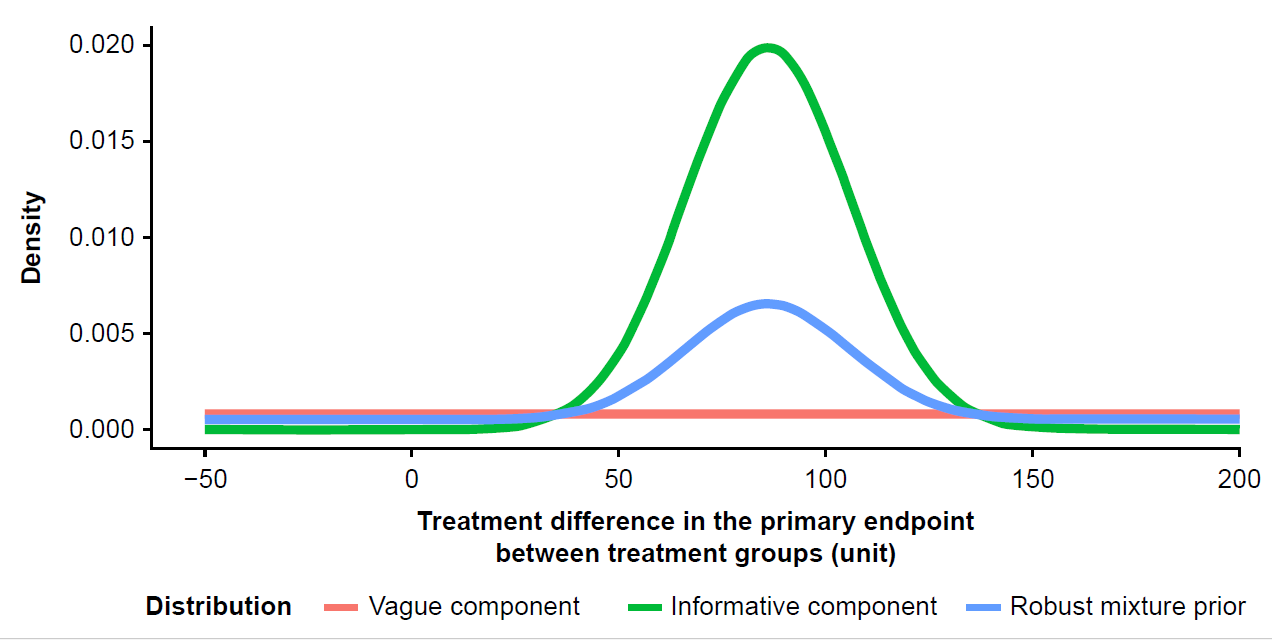


# B. Operating Characteristics of the Bridging Study Design

**Supplement Table 1: The key operating characteristics of the posterior distribution of the treatment difference**

| **Operating Characteristic** | **Description** |
| --- | --- |
| Prior weight | The initial weight of the informative component in the robust mixture prior distribution. |
| Posterior weight | The updated weight of the informative component in the posterior distribution after accounting for the China study data. |
| Updated estimate | The posterior mean estimate of the true treatment difference. |
| Bias | The updated (posterior mean) estimate minus the true value of the treatment difference. |
| Effective Sample Size (ESS) | The effective number of subjects’ worth of information per arm borrowed from global study. Estimated using method of moments [2] |
| Half-width of 90% credible interval | The half-width of the 90% credible interval of the posterior distribution of the treatment difference. |
| Minimum detectable difference | The smallest treatment difference that needs to be observed in the China study in order to meet the pre-specified success criteria when combined with the global study results via the Bayesian dynamic borrowing analysis. |
| Probability of success | The probability of meeting the study success rule of at least 95% posterior probability of the true treatment difference in China being >0. |
| Type I error (false positive rate) | The probability of success when there is no treatment difference. In the case study the type I error is the probability of success under the condition that the true treatment difference = 0 units. |
| Power | The probability of success when there is a true treatment difference. In the case study the power is the probability of success under the condition of the true treatment difference = 100 units. |

**Supplement Table 2: Posterior weights and effective sample size for possible observed treatment differences**

| **Possible observed treatment differences in China Bridging study** | **Posterior weight on the “informative” component in the posterior distribution** | **Effective number of subjects’ worth of information per arm borrowed from global study** |
| --- | --- | --- |
| 0 units | 0.56 | 5 |
| 49 units (MDD) | 0.75 | 114 |
| 60 units | 0.76 | 140 |
| 86 units | 0.78 | 174 |
| 100 units | 0.78 | 166 |

Note 1: Results based on using robust mixture prior with initial weight 0.3 on the global data which had an estimated treatment effect of 86 units.

Note 2: MDD, minimum detectable difference

Note 3: A posterior weight of 0.5 does not mean that we borrow 50% of the information / effective sample size (ESS) from the global study. The ESS and posterior weight are correlated but there is not a 1-to-1 mapping of the weight to the fraction of the global sample size that is borrowed

Supplement Table 2 shows that the updated weight and effective sample size (ESS) borrowed from the global study increase as the possible value for the observed China treatment difference gets more similar to the global study result, with maximal borrowing (corresponding to an updated weight of 0.78 and 174 patients’ worth of information per arm being borrowed from the global study) occurring if the observed China treatment difference coincides with the global treatment difference (i.e. 86 units). Note that the weight on the global study data does not increase to 1.0 in the latter scenario, since even though the *observed* result for China is identical to the global result, it is plausible that the true treatment differences are not identical because of chance variation in the observed data. So, this possibility is reflected by the posterior weight and ESS not borrowing the totality of data from the global study.

If no treatment difference (0 units) was observed in the China study, the weight on the global study would still increase in this scenario (because it is still plausible that the true China treatment difference is consistent with the global result and that the observed treatment difference of 0 unit was obtained just due to chance variation) but only to a weight of 0.56. This equates to only borrowing 5 effective subjects’ worth of information per arm from the global study.

**Supplement Table 3: Long-run frequentist operating characteristics of Bayesian Dynamic Borrowing (BDB) study design**

| **True mean difference (units)** | **Probability of success** | **Average half-width of 90% credible interval (units)** | **Bias (units)** | **Average effective sample size borrowed from global study (per arm)** | **Average posterior weight on the “informative” component** |
| --- | --- | --- | --- | --- | --- |
| 0 | 20% | 84 | 27.2 | 36 | 0.50 |
| 60 | 58% | 67 | 11.1 | 91 | 0.67 |
| 86 | 74% | 65 | -0.6 | 97 | 0.69 |
| 100 | 81% | 65 | -6.2 | 95 | 0.68 |

Note 1: Results based on using robust mixture prior with initial weight 0.3 on the global data which had an estimated treatment effect of 86 units.

Note 2: A posterior weight of 0.5 does not mean that we borrow 50% of the information / effective sample size (ESS) from the global study. The ESS and posterior weight are correlated but there is not a 1-to-1 mapping of the weight to the fraction of the global sample size that is borrowed

Supplement Table 3 shows that the BDB design has 81% power to detect a true treatment benefit in the Chinese population if the true treatment effect is 100 units, and a 20% probability of a false positive result if the true treatment effect in Chinese population is null (0 units) – the latter probability reflects that fact that a small amount of information (equivalent, on average, to an effective sample size of 36 patients) is still borrowed from the global study in the null scenario. This compares with 55% power based on a stand-alone analysis (no borrowing of global study information) of the China study data with one-sided type 1 error rate of 5% (see Figure 4 main paper). The higher type 1 error of the BDB design is consistent with the expected impact of the Bayesian framework (see e.g. Pennello and Thompson, 2008) and should be considered within the context of the bridging approach, which is proposed because of the expected similarity of the treatment difference in Chinese patients and the global population supported by similarities in clinical management of patients and consistency of treatment differences across key demographic factors including ethnicity. Calculation of operating characteristics such as those reported in here provides a transparent evaluation of the degree to which inclusion of the prior data could be beneficial or disadvantageous, to help stakeholders determine the utility of a Bayesian design in a given setting.

**Supplement Figure 2: Posterior weights of the “informative” component in the posterior distribution**

The posterior weight on the “informative” component of the mixture prior was assessed for each value of the initial prior weight (w) specified for the “informative” prior component, ranging from 0 to 1, for varying true treatment differences. Posterior weights are higher than the prior weights across the entire range of prior weights, indicating that, on average, the observed data in each scenario provide evidence supporting the relevance of the global study results for the China treatment difference. However, the increase in posterior weight is much less in the scenario where the true treatment difference in China is 0 than in the other scenarios, since the evidence of relevance is much weaker for this scenario. It may seem surprising at first that even data generated under the null scenario would provide some support, on average, for borrowing from the global study. However, this is because the sampling variability of the observed data means that the BDB model cannot completely rule out that the differences between the observed China data and the global prior information are by chance, and hence that the global prior information is relevant.


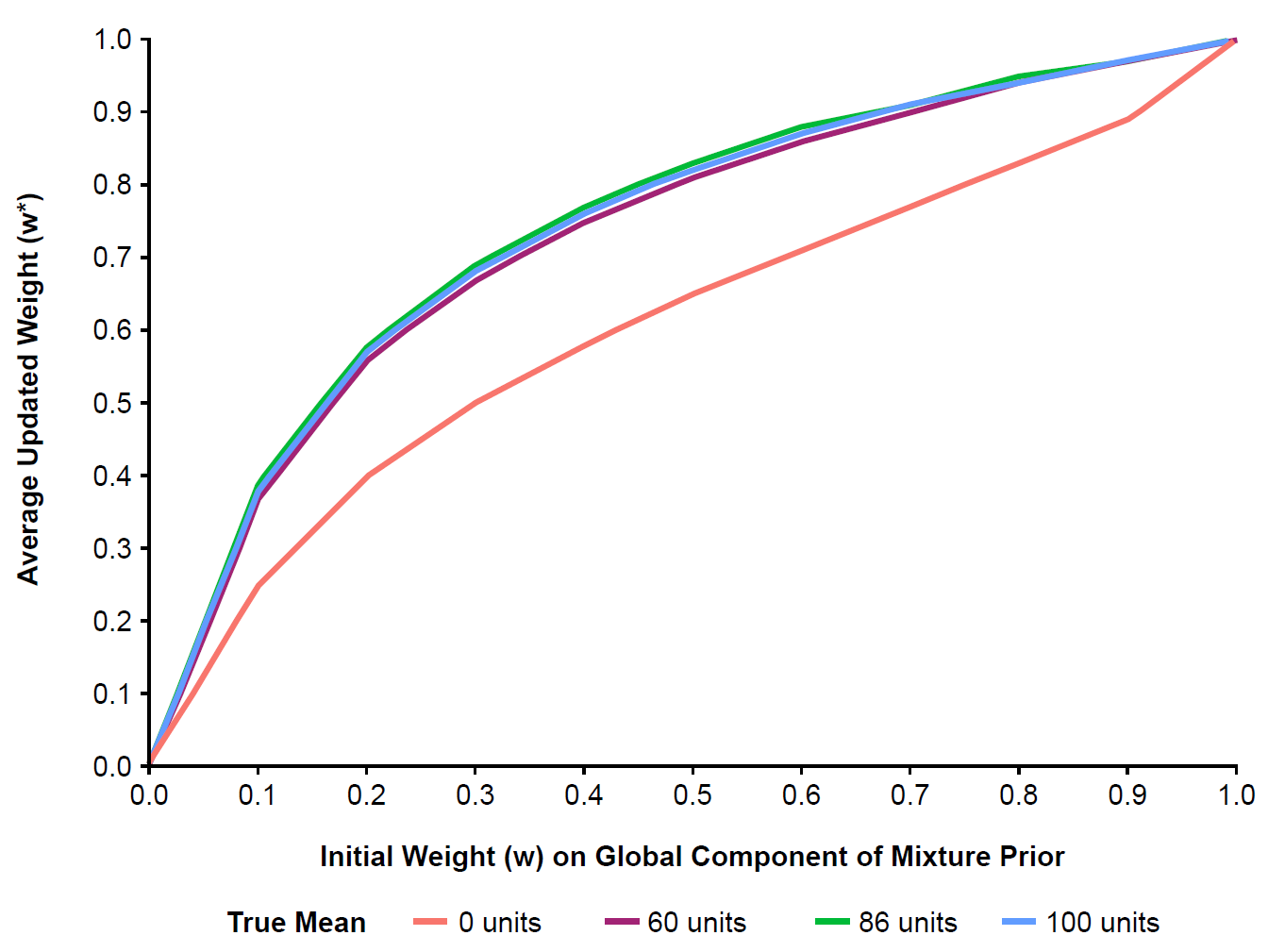


**Supplement Figure 3: Average effective sample size of the robust mixture prior**

The pattern of average effective sample size (ESS) was assessed for each value of the initial prior weight (w) specified for the “informative” component of the mixture prior, ranging from 0 to 1, for varying true treatment differences. The pattern of average ESS values across prior initial weights mirrors that seen with the posterior weights, since higher posterior weights results in more of the global study information being borrowed and hence a higher ESS.


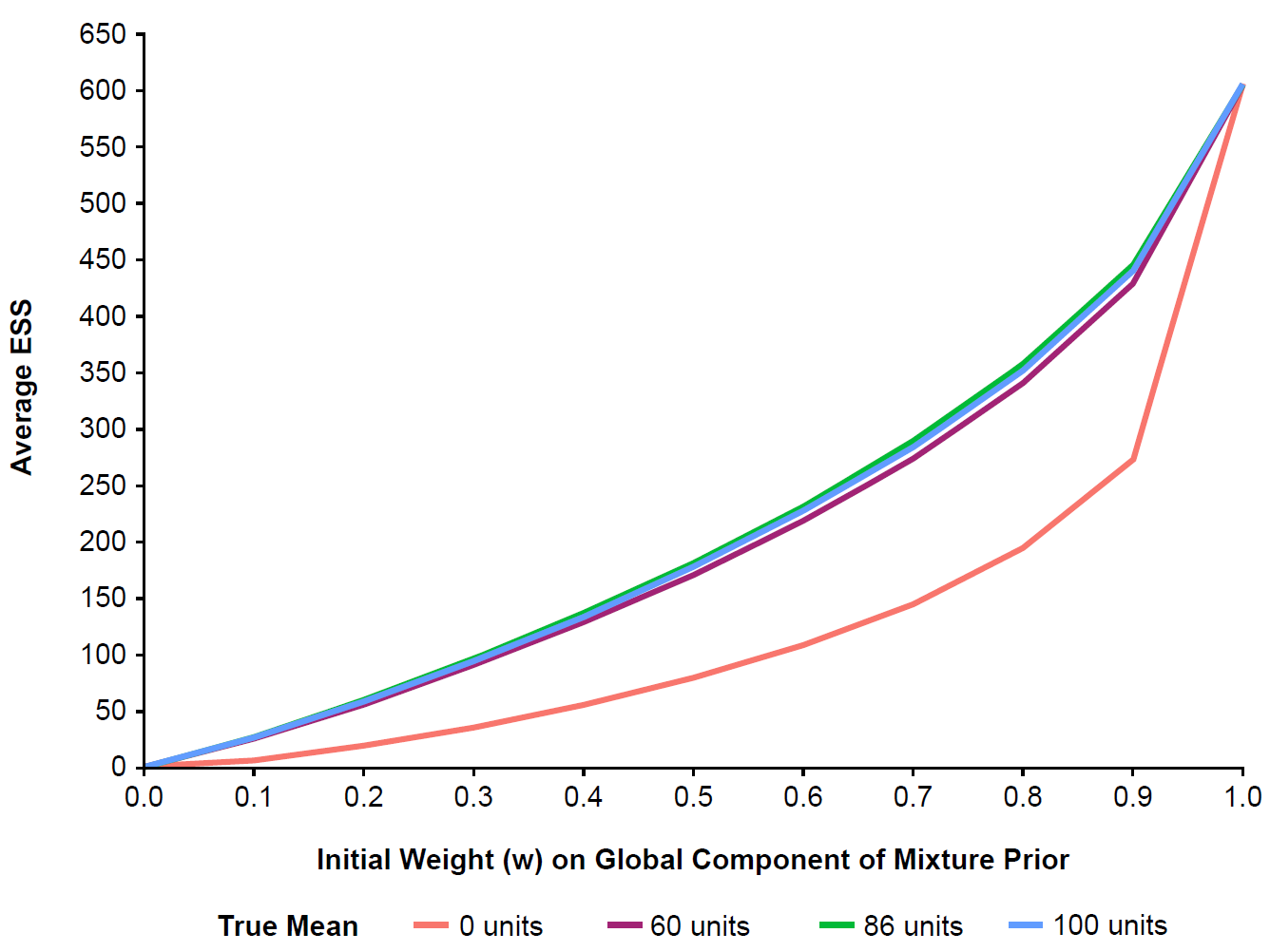


**Supplement Figure 4: Average half width of 90% credible interval**

The average half width of the 90% credible interval for the posterior estimate of the treatment difference in China was assessed for each value of the initial prior weight (w) specified for the “informative” component of the mixture prior, ranging from 0 to 1, for varying true mean treatment difference. Larger values of the initial weight $w$result in narrower credible intervals for a given value of the true mean treatment effect, reflecting the added precision gained by a more informative prior. The precision gained also depends on the true treatment effect and mirrors the pattern seen for the posterior weight and ESS – greater borrowing and hence more precision for scenarios where there is stronger evidence of relevance of the global study results for the China treatment difference.


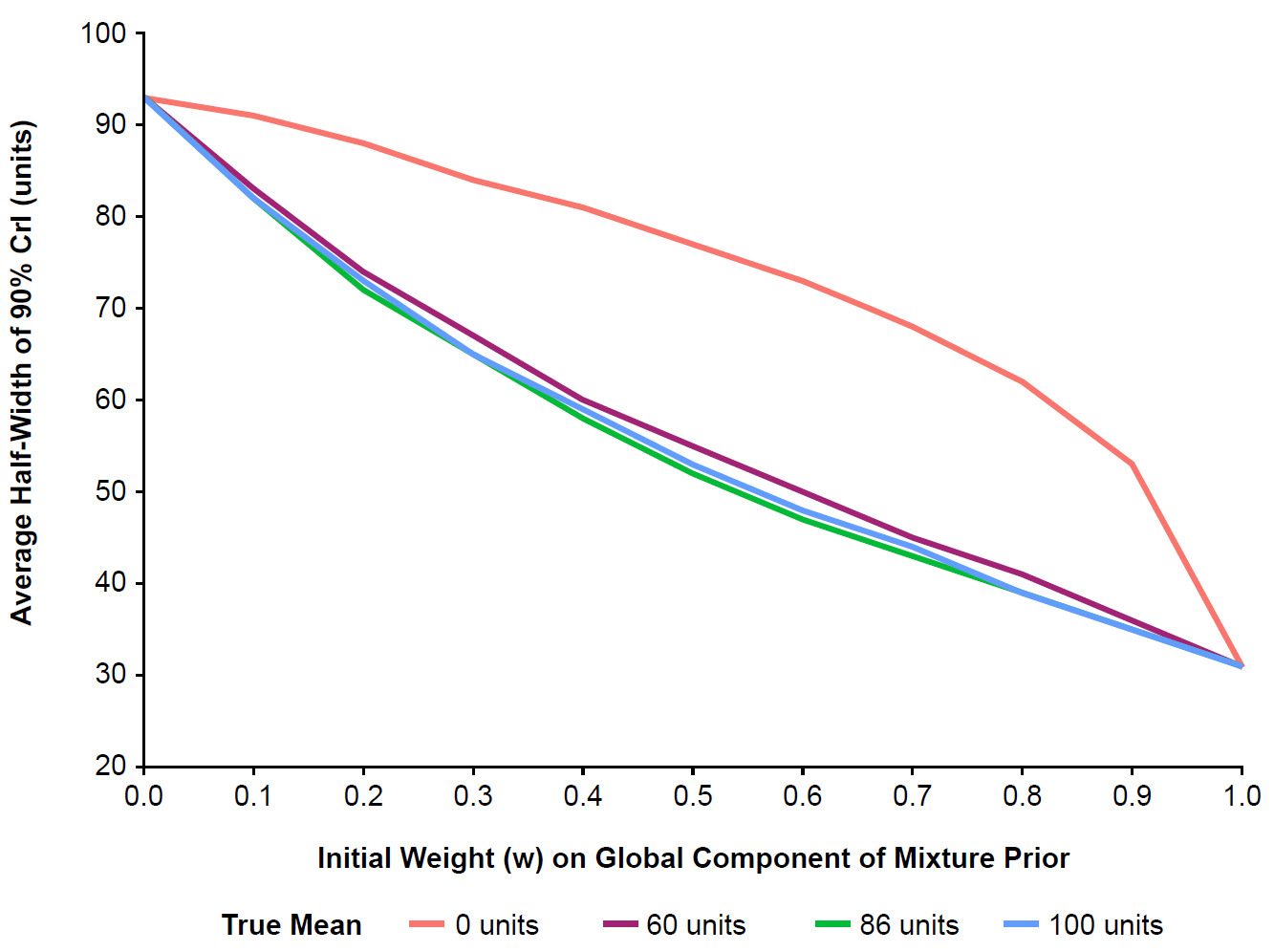


Crl, credible interval

**Supplement Figure 5: Bias across prior initial weights and true treatment differences**

The bias was assessed for each value of the initial prior weight (w) specified for the “informative” component of the mixture prior, ranging from 0 to 1, for varying true treatment difference. If the true treatment difference in China is equal to the global study result, the bias is negligible across the range of prior weights and is also small for initial weights below 0.5 for the other non-null scenarios. For the null scenario (treatment difference =0), the bias increases more sharply as the initial prior weight increases, but remains below clinically important treatment differences in magnitude (~40 units) for initial weights less than about 0.6.


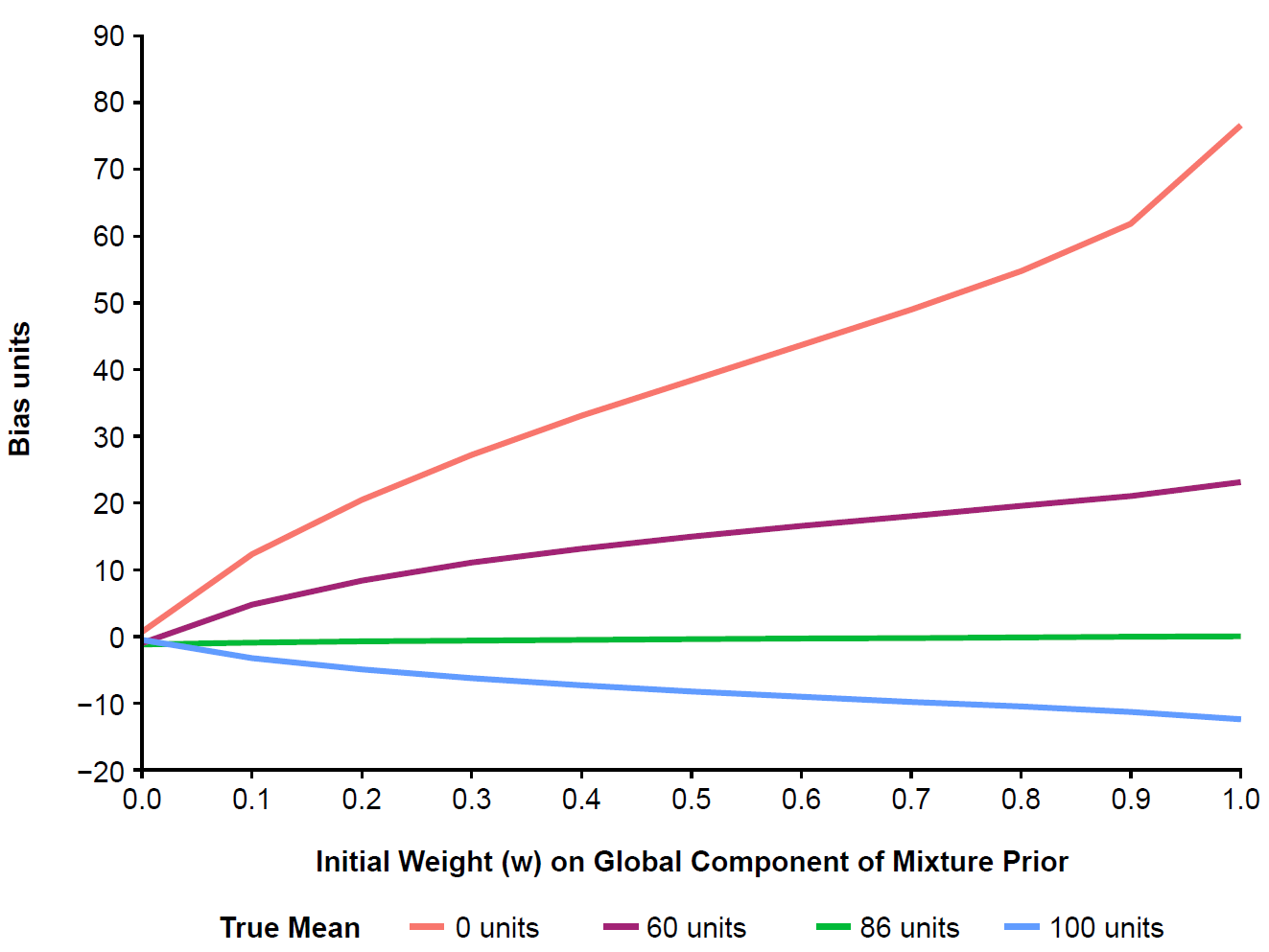


**Supplement Figure 6: Minimum detectable difference that meets the study success criteria**

The minimum detectable difference for the treatment difference in the China bridging study was assessed as a function of the value chosen for the initial prior weight (w) specified for the “informative” component of the mixture prior, ranging from 0 to 1. The proposed initial prior weight of 0.3 corresponds to observing more than 50% of corresponding treatment difference seen in the global study.


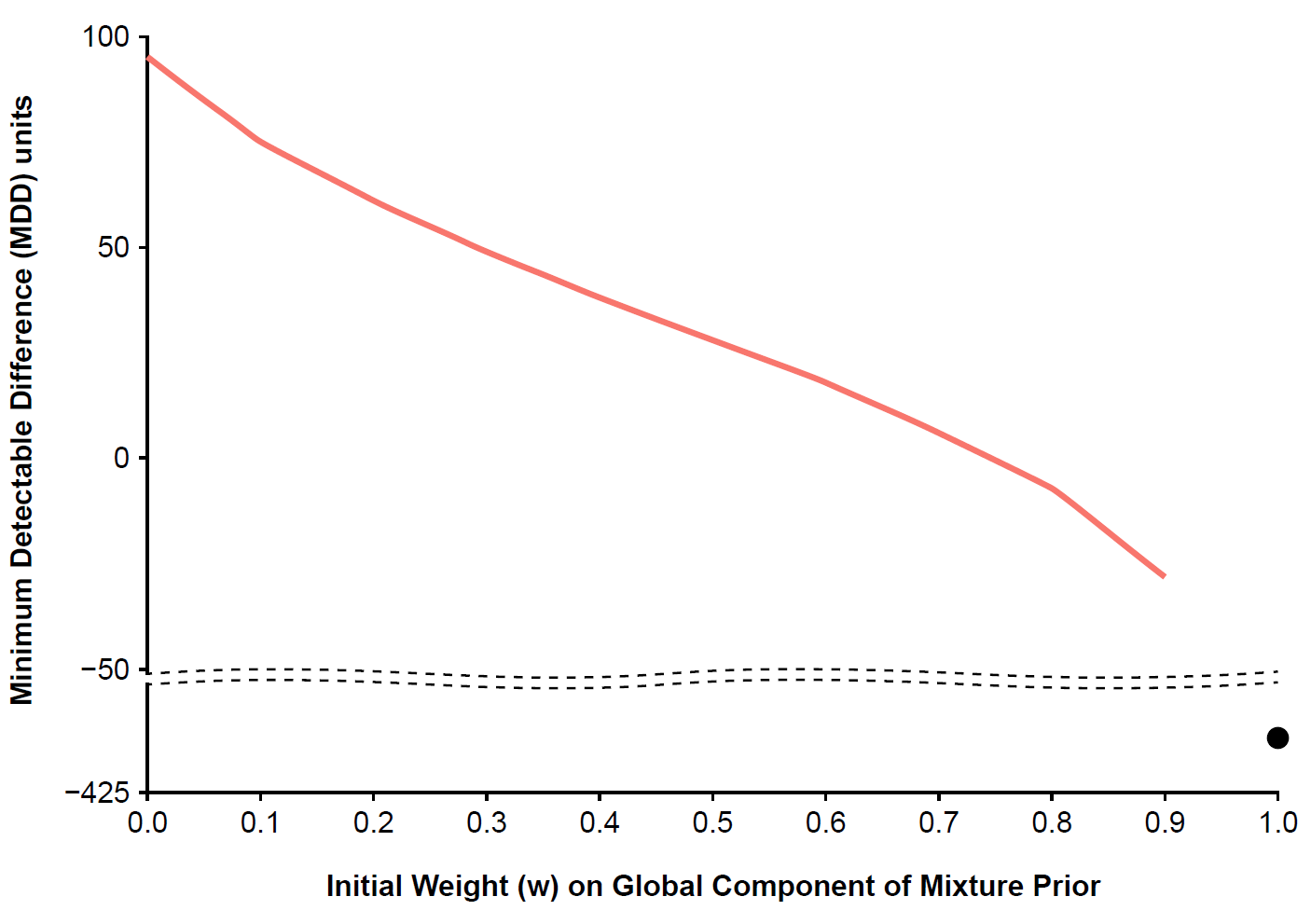


# References

1. Schmidli H, Gsteiger S, Roychoudhury S, et al. Robust meta-analytic-predictive priors in clinical trials with historical control information. Biometrics. 2014;70:1023–32.

2. Weber S, Li Y, Seaman III JW, et al. Applying meta-analytic-predictive priors with the R Bayesian evidence synthesis tools. J Stat Softw. 2021;100:1–32.
